# The role of colour patterns for the recognition of flowers by bees

**DOI:** 10.1098/rstb.2021.0284

**Published:** 2022-10-24

**Authors:** Natalie Hempel de Ibarra, Susanne Holtze, Cornelia Bäucker, Philipp Sprau, Misha Vorobyev

**Affiliations:** ^1^ Centre for Research in Animal Behaviour, Department of Psychology, University of Exeter, Exeter, UK; ^2^ Neurobiology, Institute of Biology, Freie Universität Berlin, Berlin, Germany; ^3^ Department of Optometry and Vision Science, University of Auckland, Auckland, New Zealand

**Keywords:** insect vision, behaviour, learning and memory, cognition, pollination

## Abstract

Bees discriminate between many different colours of flower petals, but it is not well understood how they perceive and learn patterns frequently found in flowers with colourful structures. We used multi-spectral imaging to explore chromatic cues in concentric flower patterns as they are seen through the low-resolution eyes of the honeybee. We find a diversity of colour combinations, which suggests that plants might exploit the sensory capabilities of pollinators, like bees, that learn colours easily. A consistent feature is that the surround of the pattern has a stronger chromatic contrast to the foliage background than the centre. This can potentially facilitate the fast identification of floral objects within colourful scenes when a foraging bee moves through a flower patch. In behavioural experiments we trained and tested bees with three types of concentric patterns. They recognized and discriminated patterns accurately in most tests, relying flexibly on both chromatic and spatial cues. Only rarely, depending on the training stimulus, chromatic cues determined their choices whilst pattern cues were ignored. The variability of floral designs and the bees' flexibility in recalling colour and spatial information suggest a role for colour vision in pattern processing. Implications for the signalling strategies of flowers are discussed.

This article is part of the theme issue ‘Understanding colour vision: molecular, physiological, neuronal and behavioural studies in arthropods’.

## 1. Introduction

Honeybee vision has been studied extensively, including their excellent colour vision. A fundamental feature of the latter is the equal spacing of three photoreceptors across the spectrum [1], from short wavelengths in the UV to long wavelengths in the near red, a highly conserved pattern among hymenopteran insects [2]. Colour-opponent neurons code for a large number of colours which significantly exceed the natural range of flower colours [3]. Since bees are completely dependent on flowers as food sources across all stages of their life, they easily memorize colours and other floral cues. In turn, insect-pollinated plants have evolved flowers of diverse colours to advertise themselves and to be distinguished from their competitors, in order to enhance pollen transfer. Spectral measurements of petal reflectances and colour vision modelling have shown that flower colours are more discriminable for bees than for animals that do not pollinate flowers, such as primates and humans [4]. This supports the idea that pollinator-mediated selection has shaped the appearance and diversity of angiosperm flowers. There is little evidence, however, for coevolution between bees and flowers in colour signalling. On one hand, colour vision in bees and other hymenopteran is generalized and conserved [2,3]. On the other, flowers sometimes share colours with other co-flowering plants and benefit from attracting additional pollinator visits [5,6]. Also, the costs of diversifying petal pigments and surface structures might frequently outweigh the adaptive benefits of diversifying petal colours, more so when they contribute to long-distance signals that guide pollinators towards a patch. Nevertheless, colour patterns provide a means by which flowers can vary their displays over close distances where pollinators decide to initiate a visit and are also able to spatially resolve a flower's pattern and colour cues.

Indeed, often the large and small structures in flowers, from ornaments such as nectar guides to conspicuous filaments and anthers, differ in their colour from petals. This is easily observed by humans, and in most cases will also be perceived by insects as multi-coloured. Such flower displays frequently consist of a central and a surrounding part forming a concentric pattern [7–9]. In some, petals are bicoloured, having a UV-absorbing and a UV-reflecting part, which is invisible to humans but generates a contrast line that can facilitate the insect's interactions with the flower. For example, it has been shown that bees can use such lines for orienting responses after landing [10]. It is generally accepted that patterns in floral displays are functionally important for pollinators, guiding their approach, landing and orientation on the flower (e.g. [11–18]); however, little is known about when and how insects rely on their colour vision to detect and learn flower patterns [19,20]. We approach this question by extracting and analysing chromatic cues from colour patterns of natural flowers as seen through the eyes of the honeybee, and by testing how bees discriminate and recognize two-coloured concentric patterns in behavioural experiments.

Pattern vision in bees has been studied mostly with black–white patterns, and there is compelling evidence that bees complete spatial tasks and discriminate pattern features through an achromatic visual system that is mediated by the L-receptor and segregated from colour vision (reviewed by [18,21]). The spatial resolution for objects such as flowers is low in bees, since receptor signals are pooled across several ommatidia; furthermore, the acuity for chromatic stimuli and patterns is lower than for achromatic visual targets [22,23]. This influences how patterns are perceived at different distances, beyond the limits set by the optics of the compound eyes [24]. On the other hand, bees learn all colours very fast, after one or a few rewarded trials [25,26], and we have previously shown that two-coloured patterns are discriminated through chromatic cues when subtending large visual angles [19,23]. Furthermore, bees and other insects have colour constancy, a sophisticated perceptual mechanism, that operates across spatial colour gradients and contrasting colour edges in visual scenes [27,28]. Taken together, this suggests that pattern information can be processed by chromatic mechanisms, and we propose that colour vision is critically involved in the identification and recognition of flower patterns.

Our previous analysis of concentric flower displays when viewed through the bee eye found that the brightness patterns of smaller-sized flowers are suited to enhance their detectability over a longer distance, which maximizes their chances of being approached and recognized from a closer distance [29]. Here, we extend this work and ask how flowers combine colours in their concentric patterns to provide chromatic cues. We find that, from short distances, when the subtended visual angle of individual flower displays is above the spatial resolution threshold of colour vision, flower patterns provide distinguishable chromatic cues. We also aim to understand how flower patterns are learned and recalled through chromatic cues. Our previous findings in discrimination experiments hint at the possibility that bees do not always memorize every aspect of the pattern of a flower that they visit [18,19]. In the present study, we show that both chromatic and spatial pattern cues influence how bees choose between learned and novel patterns and colours in discrimination and recognition tests.

## 2. Methods

### (a) Flower imaging

Multi-spectral images of freshly collected flowers of European plant species were recorded under diffused natural daylight illumination. A UV-sensitive CCD camera (Proxitronic, Germany), equipped with a quartz macrolens (105 mm, UV-Nikkor, Nikon, Japan) and five selected chromatic filters (Schott, Germany), was positioned in neutrally shadowed, north-facing locations [29–32]. Flowers were collected and transferred to the laboratory shortly before measurements started. Species were selected that displayed colourful structures in the middle of the flower and that differed notably in coloration or had different phylogenetic origins. In total, flowers from 109 European plant species from 34 families and 22 orders [33] were included in the analysis. Intact flowers with their pedicel and calyx were carefully mounted on a black vertical screen and attached with warm dental wax. Next to the flower, a scale of 12 achromatic and four coloured tiles was displayed to account for camera nonlinearities and for illumination conditions.

**Figure 1.**
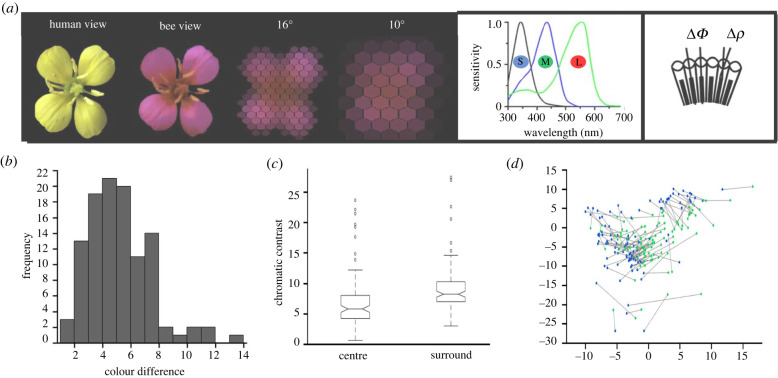
Variations of colour in the centre–surround patterns of natural flowers. (*a*) A flower image at full resolution (human and bee receptor view) and as seen through the honeybee eye when the flower subtends visual angles of 16° and 10°. These angular sizes are close to the thresholds of spatial resolution of the chromatic and achromatic visual system in honeybees. Each hexagon indicates an ommatidium. The middle panel shows the spectral sensitivity curves for the three photoreceptor types of the honeybee eye (S, short-wavelength; M, middle-wavelength; L, long-wavelength receptor; sometimes also termed UV, blue and green receptors) [1]). (*b*) Distances between the colour loci of the centre and the surround in flower patterns were well above the discrimination threshold [34]. (*c*) Chromatic contrasts of the centre and surround of flower patterns against an average foliage background. (*d*) Colour loci of the centre (green dots) and surround (blue dots) in a flower pattern in the honeybee colour space (receptor-noise limited (RNL) model [34,35) relative to the locus of an average foliage background 00).

Quantum catches, *Q*_*i*_*,* of the honeybee receptors (*i* *= S,M,L*) were calculated for each point of the image as a linear combination of the camera signals (RGB), *A_k_*:

2.1 $$Q_{i}=\sum_{k}C_{ik}A_{k},$$

where *C*_*ik*_ are coefficients with values depending on the spectral sensitivity of the honeybee photoreceptors (figure 1), on statistics of flower spectra and on the daylight illumination spectrum [30].

To simulate the optical resolution of the pattern from a viewing distance at which a flower subtends 16°, each image was projected onto the honeybee compound eye in a computer-based model [30]. The following optical parameters describe the ommatidial lattice and curvature in the frontal part of the honeybee eye: the horizontal and vertical interommatidial angles Δ*Φ*_V_ = 0.9° and **Δ*Φ*_H_ = 1.6° and the acceptance angle of an ommatidium (Δ*ρ* = 2.6°) [36–38]. We then calculate the total quantum catch the total quantum catch, *Q*^om^, for each ommatidium of the lattice, as

2.2 $$Q^{\mathrm{om}}=\int \int A(\Phi_{V},\;\Phi_{H})\;Q(\Phi_{V},\;\Phi_{H})\;\cos\beta \;d\Phi_{V}\;d\Phi_{H},$$

where *Φ*_H_ and *Φ*_V_ determine the angular coordinates relative to the centre of the visual field of the ommatidium in the vertical and horizontal directions. The quantum catch *Q*(*Φ*_V_, *Φ*_V_) is computed for each point of the image, and *β* is the angle with respect to the normal to the image plane. The angular sensitivity function, $A(\Phi_{V},\;\Phi_{H})$, of an ommatidium [39] is

2.3 $$A(\Phi_{V},\;\Phi_{H})=\exp\left[-2.77\left(\frac{\Phi_{V}{\;}^{2}+\Phi_{H}{\;}^{2}}{\Delta \rho^{2}}\right)\right].$$

Colour loci of the centre and the surround in flower patterns and chromatic contrasts to an average foliage [29,40] were determined from an ommatidium's quantum catches using the receptor-noise limited (RNL) model of bee colour discrimination [34].

### (b) Learning of concentric colour patterns

#### (i) Set-up and stimuli

Freely foraging honeybees, *Apis mellifera* (Buckfast), from a nearby apiary were recruited to an artificial grey feeder that was placed outside a large, north-facing window on the ground floor of a laboratory building. The feeder contained 30% sucrose solution (w/w), and single bees arriving at the feeder were selectively trained with 50% sucrose solution to visit a Y-maze (described below). The apparatus was located directly at the large open window and illuminated by natural daylight (see also [41]).

We also tested foragers that did not have prior experience with flowers and their colourful displays. Newly hatched honeybees were transferred from an incubator to a two-frame queen-right observation hive inside a flight cage (1 m^3^) that was situated in a glasshouse. When the bees matured and started to forage, they were only able to visit grey feeders inside the flight cage, therefore being colour-naive at the start of the experiment (see also [42]). The flight cage was connected to a second flight cage with a tunnel with doors that were manually opened to allow individually marked foragers to fly through and visit a Y-maze of the same type and dimensions as described below. The flight cage had long two-way zippers that provided easy access to the Y-maze. Both in the outdoor and glasshouse experiments, bees were allowed to return to their hives to unload the collected sucrose solution.

Each Y-maze was covered with UV-transparent Plexiglass. Only one bee was present at any time inside the Y-maze. After a bee had entered the Y-maze it could see the stimuli presented on the two backwalls and decide which arm to enter. The backwalls were placed at a distance of 15 cm from the centre of the decision chamber. At this distance, the circular stimuli (8 cm in diameter) subtended a visual angle of 30°, which is well above the chromatic detection threshold of 15° for single-coloured stimuli and concentric colour patterns [22,23]. Within this range, the bee's sensitivity for the achromatic L-receptor contrast is low, and it relies predominantly on chromatic information [24,43–45]. Nevertheless, we also selected colour pairs for the patterns that had the same or nearly identical L-receptor contrasts to avoid the use of achromatic contrast (table 1).

**Table 1.** Chromatic properties of pattern colours. Each row shows spectral variables for each of two colours that could appear in either the centre or surround of a pattern. The colours paired in a pattern (left column) were distinguishable to bees, as their colour loci were located in different parts in the colour space and separated by a large distance (chromatic distance measured in RNL units, shown in the second column [34]). Some colours differed more strongly in their chromatic contrast to the grey background than others (third column). Each colour pair was matched in their L-receptor (achromatic) contrast (right column), with small variations that were well below the sensitivity threshold, in order to remove the influence of achromatic cues [19,45–47]. The chromatic and achromatic stimulus values for naive bees that were tested inside a glasshouse, under slightly different natural daylight illumination, are shown in parentheses.

colour pair	chromatic distance	chromatic contrast to background	difference between chromatic contrasts	L-receptor contrast
yellow/cyan	16.64 (16.27)	13.99/3.66 (14.06/3.34)	large	3.97/3.87 (3.96/3.88)
green/blue3	13.16	10.18/4.28	large	2.13/2.18
orange2/green2	5.60	8.41/5.58	large	1.38/1.48
orange/green	4.99	14.82/10.18	large	2.34/2.13
blue/brown	13.74	7.06/6.68	small	1.18/0.90
violet/brown	13.54	7.48/6.68	small	0.89/0.90
blue2/green3	9.71 (9.68)	6.35/6.27 (5.90/5.83)	small	1.64/1.65 (1.65/1.65)
blue/violet	5.25 (4.26)	7.06/7.48 (6.69/7.38)	small	1.18/0.89 (1.19/0.90)

Stimuli were cut out from graphic design paper (HKS-N, K + E Druckfarben, Germany). The maze backwalls were covered with a dark grey paper (HKS 92N), as in our previous experiments [22–24,46,48]. In table 1, we list the pairs of colours that were combined to generate chromatic patterns. We used colour papers that to the human observer were yellow (HKS2N), orange (HKS6N, 8N), cyan (HKS 50N), blue (HKS 43N, 44N, 40N), violet (HKS 33N), brown (HKS 82N) and green (HKS 54N, 57N, 60N).

Reflectance spectra of the colour papers and the illumination inside the glasshouse were measured with a photospectrometer (Ocean Optics, Florida, USA) (electronic supplementary material, figure S1). Quantum catches and chromatic contrasts were calculated as described above (see also [23]). To determine receptor-specific contrasts, all quantum catches were normalized to the grey background (HKS 92N).

Colour loci in the bee colour space, chromatic distance as the distance between them and chromatic contrast to an average foliage background were then determined using the Receptor Noise Limited model (see also [23,34]). Receptor-specific contrasts (*q_i_*) were calculated as

2.4 $$q_{i}=\frac{Q_{i}^{t}}{Q_{i}^{b}},$$

where $Q_{i}^{t}$ and $Q_{i}^{b}$ are the quantum catches of receptor *i* corresponding to target and background colours, respectively.

$$Q_{i}=\int_{300}^{700}I(\lambda )S_{i}(\lambda )R(\lambda )\;d\lambda ,$$

where *i* = *S*,*M*,*L*; *λ* denotes the wavelength, *I*(*λ*) is the illumination spectrum (standard function D65 [49]), *S_i_*(*λ*) is the spectral sensitivity function of receptor *i* [1], and *R*(*λ*) is the reflectance spectrum of the coloured paper. The distance in the colour space (chromatic distance) was calculated as

2.6 $$\Delta S=\sqrt{\frac{\omega_{S}{\;}^{2}{\left(\Delta f_{L}-\Delta f_{M}\right)}^{2}+\omega_{M}{\;}^{2}{\left(\Delta f_{L}-\Delta f_{S}\right)}^{2}+\omega_{L}{\;}^{2}{\left(\Delta f_{S}-\Delta f_{M}\right)}^{2}}{{\left(\omega_{S}\omega_{M}\right)}^{2}+{\left(\omega_{S}\omega_{L}\right)}^{2}+{\left(\omega_{M}\omega_{L}\right)}^{2}}},$$

where *ω_i_* denotes the standard deviation of the noise in the receptor mechanisms *i*, *f_i_ =* ln *q_i_* is the receptor signal and *Δf_i_* the difference in receptor signals between two stimuli. The *ω*_*i*_-values were obtained from electrophysiological recordings in single photoreceptor cells [34]. According to this estimate *ω*_*S*_ = 0.13, *ω*_*M*_ = 0.06 and *ω*_*L*_ = 0.12. Equation (2.3) defines *ω_S_* so that the unity distance corresponds to one standard deviation of the noise.

#### (ii) Pre-training

Both freely foraging (henceforth experienced) and similarly colour-naive honeybees were pre-trained over 2–4 visits to locate the reward (50% sucrose solution, w/w) on the assigned colour pattern located in the centre of the left or right grey backwall. The solution was dispensed from a transparent, back-filled pipette tip that slightly protruded out of the front of the backwall [22,23]. It was only presented during training trials. Bees were individually marked, and only one bee was allowed to enter the maze at a time.

#### (iii) Experiment 1: learning of colour patterns with varying chromatic contrasts

Bees received a training of 10 trials during which they had to choose the arm with the training pattern in order to be rewarded. The backwall in the alternative arm was unrewarded and displayed only the grey background. The side of presentation was pseudo-randomly switched between visits. Both colours covered the same area in the centre (5.8 cm in diameter) and surround of the pattern (8 cm outer diameter). The reward was located in the centre of the pattern and only presented during training trials.

After completing the training phase, bees were presented with two unrewarded single-coloured discs (8 cm in diameter) during an unrewarded test. The bee's choices were observed for 2 min and audiotaped by the experimenter. After the test, the bee completed two training trials before returning for another test in which the same stimuli were presented but the side of presentation was swapped. The order of side presentation was varied between bees, and stimuli were frequently renewed. In one group (trained to the pattern with brown-centre and violet-surround), bees had another test before the single-colour test.

#### (iv) Experiment 2: discrimination and recognition of colour patterns

Experienced honeybees were trained to either a single-coloured disc or a disc displaying a two-coloured pattern. All stimuli measured 8 cm in their outer diameter. A training pattern was either an equal-area pattern (as in Experiment 1), a dot pattern (central dot of 1 cm diameter) or a ring pattern (ring with 7 cm inner diameter). As above, each bee was trained to only one stimulus. Two colour pairs were selected, yellow–cyan and blue–violet (table 1), which had a different or the same chromatic contrast against the background, respectively.

To test learning and identify recognition strategies, we conducted unrewarded 2 min test trials in which bees were confronted with fresh stimuli displaying the three patterns or single colours. Each bee was tested with four sets of test stimuli, and choices were recorded as contacts with the pattern. Each test was repeated, swapping the side of presentation, and test sequences varied across bees. Training trials were interspersed (two between side changes and five between different tests) to keep the bees motivated.

Statistical analysis was conducted in Matlab and IBM SPSS. After checking whether the requirement for normality was met, paired comparisons were conducted as *t*-tests or Wilcoxon tests.

## 3. Results

### (a) Chromatic cues in flower patterns

Using multi-spectral flower imaging, we measured and characterized the spectral properties of centre–surround flower patterns in individual flowers, as seen by the bee at close distances from the flower. An image was projected onto the ommatidial lattice of the honeybee eye when subtending a visual angle of 16*°*, which is close to the acuity threshold of bee colour vision (figure 1*a*). Flowers varied in size from 0.4 to 5.6 cm (mean ± s.d. = 2.1 ± 1.3 cm, *N* = 109), which corresponds to viewing distances of 1.4–19.9 cm, respectively.

Receptor signals were extracted for ommatidia that looked at the centre or the surround of the pattern. We found that the coloration of the underlying floral structures gave rise to well-discriminable colours (figure 1*b*). Colour loci of the centre and surround were spread widely across the bee's colour space (RNL model [34,35], figure 1*d*), suggesting that flowers form a large range of diverse centre–surround colour patterns.

The majority of species had a stronger chromatic contrast to the foliage background in the surround than in the centre (88 out of 109 species, sign test, *x* = 6.322, *p* < 0.001)—a very consistent pattern despite the different underlying floral structures that formed the pattern (figure 1*c*). Thus, there was a common feature that could facilitate filtering of chromatic information for the identification of flowers as foraging targets.

### (b) Colour choices after training with an equal-area colour pattern

**Figure 2.**
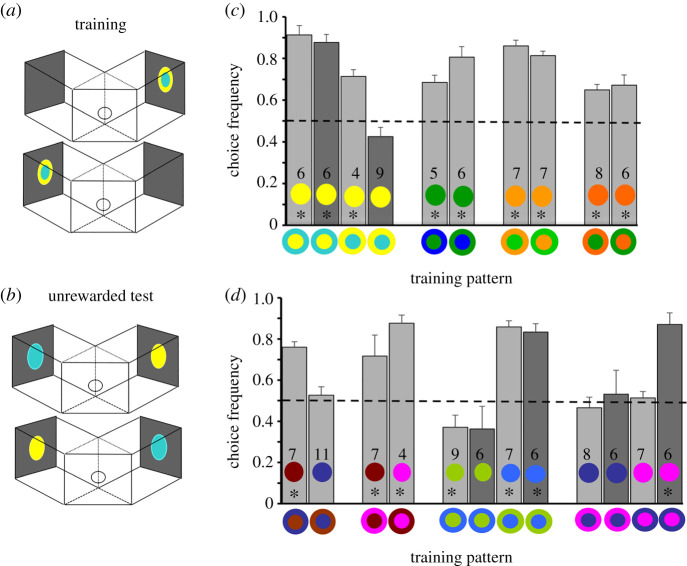
Learning of equal-area patterns (Experiment 1). (*a*) Bees were trained for 10 trials in a Y-maze. The side of presentations was varied. The backwalls were set at a distance of 30 cm. The pattern subtended a visual angle of 30° from the middle of the decision chamber, which is within the range in which chromatic cues in colour patterns are detected and discriminated [23]. (*b*) In the test, unrewarded single-coloured discs were displayed. The side of presentation was swapped and the test repeated after two refreshment trials. (*c*) Shown are the choice frequencies for the higher contrasting colour of the training pattern made by experienced (light bars) or naive foragers (dark bars). *p < 0.05; sample sizes for each group are shown inside the bars. From the right to left, patterns are arranged in decreasing order of colour similarity between the two pattern colours for bees. (*d*) The same as (*c*), except that colours did not differ in chromatic contrast against the grey background.

In Experiment 1, we trained experienced foragers and naive bees to concentric patterns that were composed of two colours and tested them with unrewarded discs displaying each colour alone (figure 2*a,b*). Both colours in a pattern occupied the same area and had similar achromatic, L-receptor contrast (table 1), so that pattern colours only differed in their chromaticity (angle) and their chromatic contrast (distance) from the grey background. We predicted that bees would approach and inspect both test stimuli equally if colour cues but not spatial cues were the main determinants in pattern learning. Alternatively, they could prefer the colour of the pattern's centre which predicts the location of the reward.

Contrary to both predictions, experienced bees showed a preference for the higher contrasting colour of the pattern in both reciprocal arrangements (figure 2*c*; electronic supplementary material, table S1; paired *t*-test/Wilcoxon, yellow-centre/cyan-surround, d.f. = 5, *t* = −5.772, *p* = 0.002; yellow-surround/cyan-centre, d.f. = 3, *t* = −3.413, *p* = 0.042; green-centre/blue3-surround, d.f. = 4, *t* = 3.038, *p* = 0.038; green-surround/blue3-centre, d.f. = 5, *t* = −5.467, *p* = 0.003; orange-centre/green-surround, d.f. = 7, *t* = 6.333, *p* < 0.001; orange-surround/green-centre, d.f. = 5, *t* = 3.806, *p* = 0.013; orange2-centre/green2-surround, d.f. = 6, *z* = −2.371, *p* = 0.018; orange2-surround/green2-centre, d.f. = 6, *t* = 10.961, *p* < 0.001). Similarly, colour-naive bees had a preference for the colour of the centre when it was yellow, which had a higher chromatic contrast to the grey background than the cyan (d.f. = 5, *t* = 3.973, *p* = 0.007). However, bees that were trained with the reciprocal pattern chose both colours equally in the unrewarded test (d.f. = 5, *t* = −1.694, *p*= 0.129). This suggests that spatial cues influenced their responses.

For patterns in which both colours had a similar chromatic contrast to the background, the choices were more varied, in ways that suggest a combined effect of spatial and colour cues for some of the colour pairings. In three out of the four colour combinations (figure 2*d*), experienced bees either (i) preferred the colour of the centre (brown-centre/violet-surround, d.f. = 3, *t* = 3.986, *p* = 0.028; violet-centre/brown-surround, d.f. = 6, *t* = −2.653, *p* = 0.038), or (ii) preferred one of the two colours (blue2) independently of its spatial position in the pattern (blue2-centre/green3-surround, d.f. = 8, *t* = −2.311, *p* = 0.0496; blue2-surround/green3-centre, d.f. = 6, *t* = −6.66, *p* < 0.001), or (iii) chose both colours equally (blue-centre/violet-surround, d.f. = 7, *t* = 0.582, *p* = 0.579; blue-surround/violet-centre, d.f. = 6, *t* = 0.342, *p* = 0.744). In the fourth colour combination, they preferred the colour of the centre in one pattern arrangement, but in the other they chose both colours equally (blue-centre/brown-surround, d.f. = 10, *t* = −0.0793, *p* = 0.446; brown-centre/blue-surround, d.f. = 6, *t* = 9.936, *p* < 0.001). The choices of naive bees mostly resembled those of experienced bees except for one pattern (violet-centre and blue-surround), where they preferred the violet colour of the centre while experienced bees showed equal preferences.

A longer training or the presence of an unrewarded alternative, an equal-area pattern with the reversed colour arrangement, during training did not change the test results (yellow–cyan patterns, electronic supplementary material, figure S2 and table S1), showing that 10 trials of training was sufficiently long for bees to memorize the flower-like colour patterns.

### (c) Discrimination of concentric patterns with unequal areas

**Figure 3.**
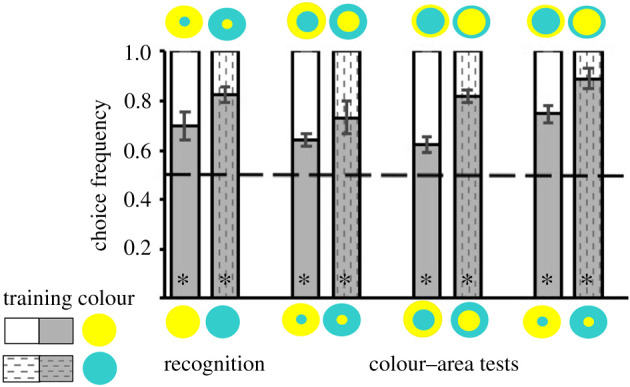
Discrimination of novel pattern. After training with either a cyan (*N* = 6 bees) or yellow disc (*N* = 7 bees), bees completed four tests. The recognition of the training stimulus was tested, presenting it against a novel pattern that differed only in the presence of a differently coloured dot. In two colour-area tests, the novel test stimuli were varied in both their spatial cues and the arrangement of colours (equal-area pattern versus reversal ring pattern, dot pattern versus reversal ring pattern). In the third colour-area test, bees chose between novel patterns that varied only in their spatial cues (dot pattern versus equal-area pattern). The colour-area tests aimed to determine whether the bees' choices were guided by the larger area covered by the learned colour. Asterisks (*) denote choices above chance with *p* < 0.05. The results of the statistical tests are given in the electronic supplementary material, table S3.

In Experiment 2, we first aimed to establish that bees are able to discriminate between yellow–cyan patterns with equal or unequal areas based on spatial cues. To this end, we trained bees with a single-coloured disc and offered them in unrewarded tests patterns that contained the learned colour and a novel colour in different proportions (figure 3). We found that bees consistently preferred the test pattern with larger content of the trained colour in each of four unrewarded tests. They correctly recognized the single-coloured training disc when presented against a pattern with a small differently coloured dot in the centre (henceforth dot pattern) (paired *t*-test, *t* = 3.107, *p* = 0.021). When presented with the dot pattern and the equal-area pattern with the same colour distribution, they preferentially chose the dot pattern, in which the learned colour covered a larger area (*t* = 3.312, *p* = 0.016). They also preferred the equal-area pattern over a pattern in which the thin surround (henceforth ring pattern) displayed the training colour and the large centre the alternative colour (*t* = 2.667, *p* = 0.037). The same was observed when the dot pattern was presented against the ring pattern with the reversed colour presentation (*z* = −2.37, *p* = 0.018). The test results clearly demonstrate that bees assessed both spatial and colour cues in the task and were not spontaneously attracted to a colour pattern. They could extrapolate the colour association when choosing between novel patterns, preferring the pattern in which a learned colour covers a larger area than the novel colour.

**Figure 4.**
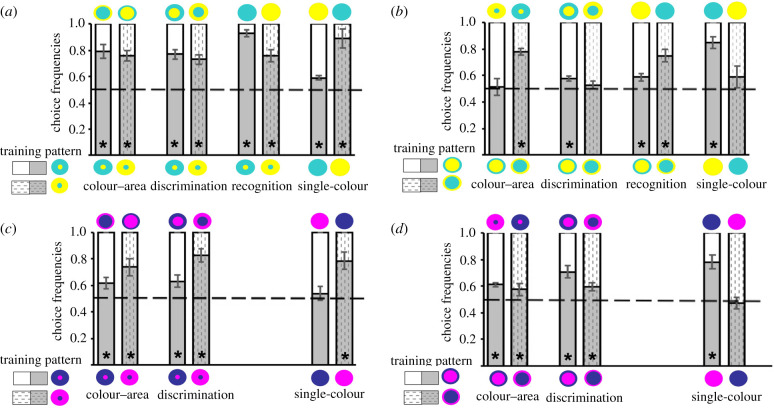
Discrimination and recognition of learned colour patterns. After training to one of eight colour patterns, bees were presented with unrewarded tests. Stimuli used in the colour-area, discrimination, recognition and single-colour tests are shown above and below each bar. (*a*) The dot pattern that bees were trained with combined two colours that differed in chromatic contrast to background (yellow-centre dot pattern, *N* = 7 bees; cyan-centre dot pattern, *N* = 6 bees). (*b*) Ring patterns were used as training patterns with the same colours as in (*a*) (yellow-centre ring pattern, *N* = 6 bees; cyan-centre ring pattern, *N* = 6 bees). (*c*) Two colours were combined that had the same chromatic contrast (violet-centre dot pattern, *N* = 9 bees; blue-centre dot pattern, *N* = 7 bees). (*d*) Ring patterns combined the same colours as in (*c*) (violet-centre ring pattern, *N* = 6 bees; blue-centre dot pattern, *N* = 11 bees). *p < 0.05 for contacts or approaches, respectively. Statistical test values are given in the electronic supplementary material, tables S4 and S5.

The tests further showed that the bees were able to perceive the spatial differences between the three pattern types. The question arose whether this discrimination could be affected when recalling a learned pattern, as it would be more similar to the novel patterns. We therefore trained new groups of bees with an equal-area patterns and tested them with novel patterns (electronic supplementary material, figure S2B,C). They clearly preferred the ring pattern over the dot pattern, which shows that they discriminated the patterns. It also suggests that the dot pattern was perceived as more distinct from the equal-area than the ring pattern (yellow-centre/cyan-surround, d.f. = 5, *t* = 3.361, *p* = 0.02; cyan-centre/yellow-surround, d.f. = 6, *t* = 2.705, *p* = 0.035; blue-centre/violet-surround, d.f. = 6, *t* = 2.858, *p* = 0.024; violet-centre/blue-surround d.f. = 7, *t* = 4.269, *p* = 0.004; electronic supplementary material, table S2). Interestingly, there was an unexpected effect as to which of the novel test patterns was preferred. Bees that were trained with a single-coloured disc (figure 3) preferred the dot pattern over the ring pattern, while after training with an equal-area pattern bees chose the ring pattern more often. This difference is striking, and while both tests show that the bees can discriminate between patterns, it is evident that preferences for novel patterns differ depending on the previously learned information.

Finally, we trained eight new groups of bees to either a dot pattern or a ring pattern displaying one of four colour combinations (figure 4; electronic supplementary material, table S5). We chose two colour pairs in which colours were easy to discriminate for bees but that had similar or different chromatic contrasts against the grey background (table 1). Since one colour covered a large area in the pattern, this could influence how bees memorize the training pattern and respond to novel patterns in a test. In the colour-area test, we conflicted the spatial cue and the colour of the larger area in the training pattern. We aimed to observe whether bees would accurately rely on the learned representation of the pattern or whether they would extrapolate one colour (as in figure 3), choosing the novel pattern equally as often as the training pattern. Similarly, in the single-colour tests, bees could show a preference for the colour that covers the larger area in a pattern if they encode spatial differences between colour elements. In the discrimination test, the novel pattern only differed in spatial cues, and in the recognition test in both colour and spatial cues. For both tests, we predicted that bees would solve the task correctly, based on the findings shown in figure 3.

After training with yellow–cyan dot patterns (figure 4*a*), bees in both groups distinguished and preferred the training pattern over each of the alternatives (all *p* < 0.05; see electronic supplementary material, table S5): the reversal ring pattern (colour-area test), the equal-area pattern (discrimination test) and a single-coloured disc of the colour that covered the larger area in the training pattern (pattern-recognition test). These results show that bees had learned the whole pattern and were able to use spatial cues to discriminate between stimuli. We also found that they had a preference for the colour that covered the larger area in the training pattern when they were presented with single-coloured discs (figure 4*a*). This is different from the findings in Experiment 1, where bees preferred the higher contrasting colour in both colour arrangements after being trained with an equal-area pattern.

Choices were less consistent in groups that were trained with the yellow–cyan ring pattern (figure 4*b*; electronic supplementary material, table S4). With regard to colour-area tests, in the group trained with the yellow-centre/cyan-surround ring pattern bees chose equally between the learned pattern and the alternative stimulus, the reversal yellow dot pattern, seemingly being guided by the large cover of the yellow colour in both stimuli and ignoring differences in spatial cues and colour arrangement (*t* < 0.001, *p* = 1.000). By contrast, after being trained with the opposite pattern (yellow-surround/cyan-centre ring pattern), bees discriminated and recognized the training pattern accurately (*t* = 4.513, *p* = 0.006). As for discrimination tests, we observed a generalization effect between the cyan-centre/yellow-surround equal-area and ring pattern (*t* = 1.214, *p* = 0.279), despite the bees' ability to spatially resolve the differences in these two patterns, as shown in the discrimination test with yellow-centre patterns and the tests after training with a single-coloured disc (figures 3 and 4*a*). Conversely, the group trained with the reversed pattern (yellow-centre/cyan-surround) discriminated the training pattern (*t* = 4.029, *p* = 0.010). Lastly, in the single-colour tests, after training to the ring pattern with a cyan-centre and yellow-surround, bees chose both colours equally (*t* = 0.391, *p* = 0.712), which is different from the findings in the same single-colour tests with yellow and cyan discs in both Experiments 1 and 2 (figures 2*c* and 3*a*), as well as the group trained with the reversed pattern (yellow-centre/cyan-surround ring pattern, *t* = 6.113, *p* = 0.002).

In figure 4*c*,*d*, we present the test data for groups of bees that were trained with blue–violet dot or ring patterns that demonstrate accurate discrimination and recognition of the learned pattern. Bees preferred the training pattern over the alternative, the reversal ring or dot pattern, respectively, which displayed a large area covered by the colour of the training pattern's centre. They also discriminated the learned pattern against the equal-area pattern with the same colour arrangement. As in Experiment 1, bees chose equally between a violet and a blue disc in the single-colour tests, but only if the blue area was large in their training pattern. This was consistent with the predictions for a learning strategy that relied on colour cues only. By contrast, after training to patterns with large violet areas, bees preferred the violet disc over the blue disc after being trained with a pattern that had a large violet area. This is, however, consistent with the observations made in Experiment 1, where choice patterns varied within and between colour pairs. In this case, bees chose in a similar way to those tested with a blue and green disc in Experiment 1.

## 4. Discussion

We document here how bees learn and recognize concentric patterns through chromatic cues. In Experiment 1, we found that bees consistently attended one colour more if it presented a higher chromatic contrast to the background (figure 2). By contrast, if the two colours did not differ in the strength of their chromatic contrast, the bees' responses were variable, which could be due to the combination of spatial and other colour cues, but seemingly is also due to their previous experience with natural flowers. Results from Experiment 2 (figures 3 and 4) suggest that bees rely flexibly on learned spatial and colour cues when discriminating the trained stimulus from a novel pattern or choosing between them. Similarly, we found that the bees' responses to single-coloured discs changed depending on the spatial cues in the pattern they memorized. Nevertheless, when one of the pattern colours had a higher chromatic contrast, bees trained with a ring pattern tended to perform less accurately.

Two recognition strategies were identified. There was accurate discrimination of coloured patterns when bees took into account both colours and their spatial distribution. By contrast when one of the colours had a higher chromatic contrast, bees sometimes switched to a colour-based recognition strategy and ignored the pattern's spatial cues (figures 2 and 4). This is likely to impact the foraging choices of bees in different ways when they encounter familiar and novel flowers under natural conditions. Considering that flowers displaying centre–surround patterns have variable combinations of colours but their surrounds contrast more strongly against the foliage (figure 1), bees may approach and visit novel flowers that share just the colour of the surround and differ in other aspects of their preferred flower's colour pattern display.

We have previously found that bees can generalize one element of a concentric two-coloured pattern in a discrimination task [19] after being trained with an equal-area pattern. Depending on the colour arrangement, bees would either discriminate the trained pattern against a single-coloured stimulus displaying the colour of the trained pattern's centre, or not. This was independent of whether the colours that were combined in the pattern had the same or different achromatic, L-receptor contrasts to the background. We could also rule out limitations in chromatic resolution because the same bees discriminated the training pattern against an unrewarding test pattern that displayed the reciprocal colour arrangement. The effect was therefore attributed to the strong difference in chromatic contrast to the background between the pattern colours, and our present study supports this conclusion. The findings from both studies illustrate that bees can choose indiscriminately in the presence of strong chromatic cues, even though they can see and spatially resolve both pattern elements.

Interesting implications for pollinator-driven selection of visual cues in floral displays arise from our results. Flowers have evolved a variety of petal colours to be distinguishable from each other, with different types of pigments, layering and other cell and surface structures that generate optical effects, such as the reflection of UV light (e.g. [50,51]). These are costly for the plant but might be less beneficial for selectively controlling pollinator visitation rates and behaviour. This is also the most parsimonious explanation for the observation that some petal colours that bees and other pollinators could perceive do not exist or are found less frequently than others, such as UV–white petals, which have a strong chromatic contrast when seen against leaves, despite their flat reflectance spectrum [3,4,47]. Absorption of UV by colour pigments has been proposed to have protective benefits for the flower [52,53]. Some plants also benefit from similarities in their petal colours when considering the environmental conditions in which they grow, their abundance, dependence on obligate outcrossing, susceptibility to pollen clogging and fluctuating abundances of the most likely pollinator species in a habitat [5,6,54,55]. Patterns therefore might provide useful means to diversify floral displays for short-distance signalling while at the same time obtaining potential benefits from sharing colour cues with other species or colour morphs within the foraging range of pollinators. This could be an effective signalling strategy for plants that would draw in pollinators and retain them in a flower patch, given that pollinators learn the locations of flowers very quickly, forming spatial preferences and having the flexibility to decide where and when to explore and exploit flowers in a patch [56–59].

The few differences between naive and experienced foragers we have found in Experiment 1 (figure 2) tentatively suggest that previous experience with natural flowers might influence how patterns are learned and recognized. More work needs to be done to investigate these effects, but we can rule out that any responses of naive bees were impacted by unlearned colour preferences after training. This is because such preferences are very quickly overwritten by learning and exposure to rewards in bees and other insects [60–62], and their expression also varies in different experimental contexts [63–66].

We add new evidence showing that despite the lower acuity of chromatic vision in bees, they are capable of using it to discriminate patterns, most likely as a complementary mechanism to the achromatic visual pathways that are required for solving a variety of visual tasks [67]. Our results imply that colour vision contributes to the encoding and representation of multi-coloured patterns, at least within the context of flowers, when bees recognize and interact with them. Such mechanisms have a high adaptive value in that they put the excellent colour vision of bees and many other pollinating insects to a good use. There are other natural sources of colour patterns besides flower displays, such as colourful views of inflorescences, bracts, flower patches in the landsape, landmarks and other features in the landscape, that are also important to bees when they choose foraging sites and navigate between them [68–72]. How chromatic and pattern information is processed at the neuronal level remains a subject of great interest. It is likely that the colour-coding neural circuitry differs in its organization from that of mammals and other vertebrates. The edge-processing achromatic and the chromatic system appear to be strongly segregated in the retinotopically organized layers of a bee's optic lobe, and peripheral colour-coding neurons have large receptive fields [73–77]. Where and when these visual systems might interact in the insect brain is not very well understood; however, recent findings in *Drosophila*, for instance provide first anatomical and functional examples for these types of interactions, via Dm neurons in the medulla, the second optical ganglion of the optic lobe [78,79].

The influence that a colour with a higher chromatic contrast has on pattern recognition strategies is intriguing also in view of the consistent difference in the strength of chromatic contrast between the centre and the surround of flower patterns. Does this mean for bees that colours and patterns intrinsically vary in their perceptual salience with higher chromatic contrast? It is tempting to propose it, yet to prove such perceptual effect is challenging and so far has not been attempted for bees and other insects ([80,81], but see [82,83]). Chromatic contrast characterizes the discriminability of a colour from its background through its chromaticity, and it is determined in the colour space as the distance between the loci of the background and the colour stimulus. In human colour vision, loci that are further away from the neutral point are perceived as more saturated, independently from colour hue. It is unknown, however, whether this is the same in insects, and we therefore refer to chromatic cues in correspondence to the modelled values and caution against the use of perceptual terms that are inferred from human vision, such as salience or saturation. A stronger chromatic contrast improves the detectability and identification of single-coloured targets over a short-distance range, as shown in studies that compared the performance with either low or high chromatic contrasts when training bees in a dual-forced choice [45], or allowing them to fly and search among several artificial flowers [84]. There is a positive relationship as bees make fewer mistakes and find flowers faster when the chromatic contrast is higher, similar to some vertebrates [85,86], which is in line with evidence for faster pre-attentive, bottom-up processing speed which underpins colour salience found in humans [87–89].

An insect's viewing of floral patterns and use of colour signals differs in fundamental ways from how humans look at flowers and take notice of small details in a floral display. Primarily, insect eyes are only capable of resolving small floral ornaments and structures when they have come close enough to the flower. Our approach for measuring the floral signals considered the sensory constraints that the optical resolution of the bee eye imposes and the detection thresholds that dictate the relevant viewing distances. We made minimal assumptions about the various structures and floral parts that contribute to the pattern display of the flower [8,10,90] to maintain the perspective of the receiver with a low-resolution eye. While easily visible to a human observer, some floral structures and variations in petal coloration [51,91] will be indistinguishable to an approaching bee until it is close enough to hover in front of the flower, land and interact with it, and the importance of such aspects in a floral display should be empirically investigated (e.g. [92]). At longer distances, the patterns of individual flowers do not play any role in plant–pollinator communication because their displays simply merge with the background and other flowers on the same or different plants that are aggregated around them (see also [72]). Instead, such shared displays generate views with distinct long-distance colour cues that can guide an insect pollinator arriving at or moving through flower patches.

Visitation patterns of pollinators are sometimes explained in terms of attraction to floral cues, although the main goal of a pollinator for finding a flower is to extract a reward. This typically involves learning of floral signals and cues that reliably predict how and where to obtain it. Bees form individual preference for a few flowers by associating cues in floral displays with reward while ignoring or rejecting flowers of other plants [56,58,93]. From a mechanistic point of view, one question might be what information the pollinator requires for the decision to choose one flower over another, and a key visual task is to recognize a flower as an object that the insect can approach and land on, in addition to discriminating and identifying it as the preferred flower. The chromatic properties of colour patterns in flowers we describe here could provide the basis for a quick and robust mechanism for segregating individual flowers from their surroundings, which are composed of foliage and other flowers presenting as colourful blobs in the insect's field of view that quickly change in appearance as the insect moves. Such patterns provide chromatic cues to facilitate the controlled execution of a safe and energetically efficient approach flight (e.g. [18]) and are useful for close-range recognition as a floral object [72,94]. Flowers could benefit from conserving features in their colour patterns as an adaptation to some types of filtering mechanisms that are ubiquitous in insect vision and other sensory systems [29,95–100]. The identification process requires further visual information for accepting or rejecting a flower as well as for deciding where to land and how to handle it (e.g. [101,102]).

These mechanisms could potentially also drive the diversification of colour patterns and the coloration of small floral organs (e.g. [92,103–105]), depending on visual strategies such as those adopted by the bees in the present study, i.e. the learning of multiple elements of flower patterns and the generalization of strongly contrasting chromatic cues. Both strategies provide flowering plants with options to diversify if needed and to use the least costly signalling solution, effectively exploiting the receiver's sensory constraints and learning capabilities.

## Data Availability

Data are openly available from the institutional repository of the University of Exeter repository at: https://doi.org/10.24378/exe.4104 [106].
